# Treinamento Físico Reduz a Inflamação e a Fibrose e Preserva a Função e a Perfusão Miocárdica em um Modelo de Cardiomiopatia Chagásica Crônica

**DOI:** 10.36660/abc.20230707

**Published:** 2024-08-21

**Authors:** Thayrine R. Damasceno, Denise M. Tanaka, Enrico F. Magnani, Rafael D. B. Oliveira, Danielle A. G. Pereira, Ildernandes Vieira-Alves, Virginia S. Lemos, Jorge M. Cabeza, Camila G. Fabricio, Alessandra A. Resende, Dawit A. P. Gonçalves, Gustavo de Oliveira Zanetti, Eduardo E. Vieira de Carvalho, Marcus V. Simões, Luciano F. L. Oliveira

**Affiliations:** 1 Universidade Federal de Minas Gerais Belo Horizonte MG Brasil Universidade Federal de Minas Gerais, Belo Horizonte, MG – Brasil; 2 Universidade de São Paulo Faculdade de Medicina de Ribeirão Preto Ribeirão Preto SP Brasil Faculdade de Medicina de Ribeirão Preto – Universidade de São Paulo, Ribeirão Preto, SP – Brasil; 3 Hospital Israelita Albert Einstein São Paulo SP Brasil Hospital Israelita Albert Einstein, São Paulo, SP – Brasil; 4 Universidade Federal do Triângulo Mineiro Uberaba MG Brasil Universidade Federal do Triângulo Mineiro, Uberaba, MG – Brasil

**Keywords:** Cardiomiopatia Chagásica, Miocardite, Aptidão Cardiorrespiratória, Exercício Aeróbico, Imagem de Perfusão do Miocárdio

## Abstract

**Fundamento:**

A Cardiomiopatia Chagásica Crônica (CCC) é causada por um processo inflamatório induzido pelo *Trypanosoma cruzi*, que leva à miocardite com fibrose reativa e reparativa. A CCC progride com alterações de perfusão miocárdica e eventos histopatológicos que afetam a Aptidão Cardiorrespiratória (ACR).

**Objetivos:**

Avaliamos os efeitos do Treinamento Físico Aeróbico (TFA) na perfusão miocárdica e nos comprometimentos morfológicos e funcionais relacionados à inflamação e fibrose em hamsters sírios com CCC. Como objetivo secundário, analisamos as áreas de secção transversa do músculo esquelético.

**Métodos:**

Hamsters com CCC e seus respectivos controles foram divididos em quatro grupos: CCC sedentário, CCC-TFA, controle sedentário e controle TFA. Sete meses após a infecção, os animais foram submetidos à ecocardiografia, à cintilografia de perfusão miocárdica e ao teste de esforço cardiopulmonar. TFA de intensidade moderada foi realizado durante cinquenta minutos, cinco vezes por semana, por oito semanas. Posteriormente, os animais foram reavaliados. A análise histopatológica foi realizada após os procedimentos acima mencionados. O nível de significância foi estabelecido em 5% em todas as análises (p<0,05).

**Resultados:**

Animais com CCC sedentários apresentaram piores Defeitos de Perfusão Miocárdica (DPM) ao longo do tempo, Fração de Ejeção do Ventrículo Esquerdo (FEVE) reduzida, e apresentaram mais inflamação e fibrose quando comparados aos demais grupos (análise ANOVA mista). Por outro lado, o TFA foi capaz de mitigar a progressão do DPM, atenuar a inflamação e a fibrose e melhorar a eficiência da ACR em animais CCC-TFA.

**Conclusão:**

Nosso estudo demonstrou que o TFA melhorou a disfunção cardíaca, DPM e reduziu a inflamação e a fibrose em modelos de hamster com CCC. Além disso, os animais CCC-SED apresentaram atrofia do músculo esquelético, enquanto os animais CCC-TFA apresentaram a AST do músculo esquelético preservada. Compreender os efeitos da TFA nas dimensões fisiopatológicas da CCC é crucial para futuras pesquisas e intervenções terapêuticas.

**Figure:**
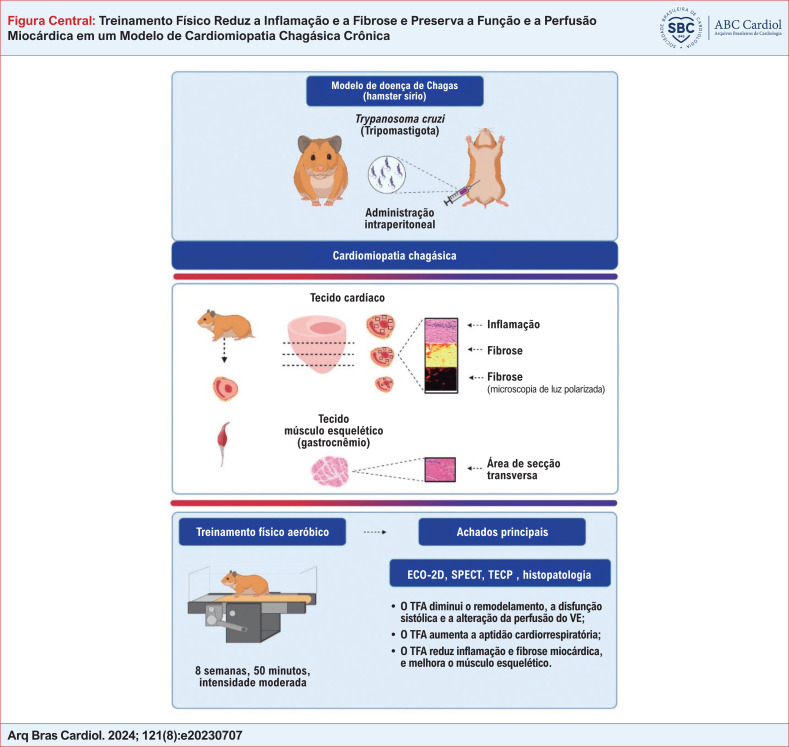


## Introdução

A doença de Chagas é uma doença tropical negligenciada que gera, anualmente, 627,46 milhões de dólares em gastos com saúde,^1^ e afeta de seis a oito milhões de pessoas em todo o mundo.^2^ A doença é causada pelo protozoário *Trypanosoma cruzi* (*T. cruzi*), e a maioria dos indivíduos infectados permanecem assintomáticos ao longo de suas vidas (forma indeterminada). No entanto, cerca de 40% evoluem para as formas clínicas (cardíaca ou digestiva) 10 a 30 anos após a infecção aguda inicial.^3^ A Cardiomiopatia Chagásica Crônica (CCC) é a cardiomiopatia não-isquêmica mais grave e progressiva na América Latina.^4^ A CCC manifesta-se com distúrbios eletrocardiográficos, alterações na motilidade e perfusão da parede, eventos tromboembólicos, e insuficiência cardíaca, o que pode levar à morte súbita.^5^

Os principais mecanismos patogênicos da CCC estão relacionados à persistência do parasita com lesão miocárdica mediada pelo sistema imune, distúrbios microvasculares e disautonomia cardíaca.^6^ Na fase crônica, a elevada carga de parasitemia observada na fase aguda é reduzida pela resposta imune para uma infecção mais leve, mas persistente.^7^ Esse processo inflamatório de baixa intensidade, leva à miocardite multifocal com infiltrados mononucleares, lesões miocitolíticas, necrose, desarranjo microvascular, hipertrofia de cardiomiócitos, áreas aumentadas de fibrose intersticial e perivascular e remodelamento cardíaco.^1,8–10^

Miocardite e fibrose reparativa são achados característicos da CCC.^6^ O infiltrado inflamatório no miocárdio é principalmente composto por células T e macrófagos.^11^ Além disso, há uma expressão aumentada das citocinas inflamatórias – Interferon-gama (INF-γ), Fator de Necrose Tumoral-alfa (TNF-α), Interleucina-6 (IL-6),^12,13^ e quimiocinas.^11,14^ A persistência do *T. cruzi*, levando à produção contínua de INF-γ e TNF-α, estimula o estresse oxidativo e nitrosativo que causa danos às mitocôndrias de cardiomiócitos e disfunção mitocondrial, e compromete vias de produção de energia.^15,16^ Além dessas alterações que estão associadas a um prognóstico ruim da doença, a disfunção microvascular coronariana e os distúrbios de perfusão miocárdica subsequentes podem ser os marcadores iniciais de progressão da doença.^17,18^ Esses distúrbios também estão relacionados à necrose miocitolítica e ao desenvolvimento de lesões cicatriciais associados a áreas de fibrose transmural regional.^19^

Além do dano miocárdico, a infecção crônica por *T. cruzi* também pode afetar o músculo esquelético. As principais alterações incluem inflamação do músculo esquelético, exsudato mononuclear,^20^ necrose das células fibrosas,^21^ desorganização e atrofia das fibras musculares,^20^ vasculite e fibrose,^22^ danos capilares com espessamento e reduplicação da membrana basal, diminuição da Área de Secção Transversa (AST) do músculo esquelético,^23^ e denervação muscular.^24^ Indivíduos com CCC também podem apresentar obstrução nos capilares e uma atividade mais glicolítica e menos oxidativa no músculo esquelético.^25^ Em conjunto, tais alterações podem afetar a extração de oxigênio, reduzir a oferta de oxigênio e causar prejuízo funcional.^25^

Um dos principais fatores associados à morbidade e mortalidade na CCC é a progressão da doença^26^ que afeta a Aptidão Cardiorrespiratória (ACR)^27^ e a qualidade de vida.^28^ Atualmente, o tratamento da CCC baseia-se principalmente no controle de sintomas da doença.^1,5,29^ Contudo, apesar da escassez de ensaios controlados randomizados,^30^ alguns estudos já observaram benefícios do treinamento físico nesses indivíduos, com melhoras importantes na ACR.^31–34^ A Diretriz mais recente sobre CCC^1^ afirma que a atividade física melhora muitos parâmetros clínicos, mas os benefícios para essa população ainda não foram completamente abordados. Assim, esta abordagem tem recomendação de grau "condicional" e um nível de evidência "B".^1^

Melhoras no VO_2_ pico foram observadas após o treinamento físico em indivíduos com CCC.^30,31^ Outros benefícios incluem melhora na fração de ejeção do ventrículo esquerdo (FEVE), força muscular respiratória,^33^ função microvascular cutânea,^35^ e qualidade de vida.^30^ Nos estudos experimentais, o treinamento físico antes e após infecção experimental por *T. cruzi* modulou a reação inflamatória e melhorou a resistência contra o *T. cruzi*,^36^ induziu redução na atividade sérica da creatina quinase e da creatina quinase forma MB^37^ e reduziu fibrose cardíaca.^38^ Na CCC, somente um estudo demonstrou que o exercício aeróbico de baixa intensidade melhora parâmetros morfológicos e morfométricos dos ventrículos direito e esquerdo.^39^

Nos estudos experimentais sobre CCC, vários modelos animais foram utilizados.^8,9,40–43^ Entre eles, o modelo murino com hamster sírio é o modelo que desenvolve CCC que lembra infecção humana com a história natural típica, e alterações histológicas, estruturais e funcionais da doença com uma linha do tempo mais adequada para estudos científicos.^10,43,44^ Apesar dessas evidências, nenhuma dessas investigações focaram nos efeitos do exercício aeróbico na perfusão miocárdica, nas alterações histopatológicas (inflamação e fibrose) e no músculo esquelético em hamsters sírios com CCC. Considerando as adaptações cardiovasculares,^45–47^ acreditamos que o exercício aeróbico pode levar a melhorias nos principais mecanismos patogenéticos envolvidos na progressão da doença: Defeitos de Perfusão Miocárdica (DPM) e inflamação. Portanto, este estudo teve como objetivo avaliar o impacto do Treinamento Físico Aeróbico (TFA) nas alterações morfológicas, funcionais e de perfusão do miocárdio, correlacionando essas variáveis com inflamação e fibrose cardíacas em hamsters sírios com CCC usando exames de imagem de alta resolução *in vivo*. Além disso, nosso objetivo secundário foi analisar as AST do músculo esquelético deste modelo experimental.

## Métodos

### Delineamento do estudo

Neste estudo experimental, hamsters sírios fêmeas (*Mesocricetus auratus*), com idade de 12 semanas, obtidos do Anilab (*Animais de Laboratório Criação e Comercio* Ltda, Paulínia/SP, Brasil) foram mantidos em uma sala com temperatura controlada com livre acesso à água e alimentação padrão, e submetidos a um ciclo claro-escuro de 12 horas. Os animais foram mantidos em uma gaiola seguindo-se as recomendações do CONCEA (Conselho Nacional de Controle de Experimentação Animal), com uma área por animal > 122,5 cm^2^ e enriquecimento ambiental apropriado para a espécie. Todos os procedimentos foram conduzidos durante a fase clara do ciclo claro-escuro.

Os animais foram aleatoriamente alocados usando uma ferramenta de randomização (www.random.org Random.org, Dublin, Irlanda). Inicialmente, os animais (n=60) foram alocados em grupos infectados e não infectados. Após a fase aguda da infecção, os animais sobreviventes [infectados (n=37), não infectados (n=16)] foram separados em quatro grupos experimentais [TFA e sedentários: CCC-TFA (n=22), CCC-SED (n=22); e dois grupos controles também separados em TFA e sedentários: CT-TFA (n=8) e CT-SED (n=8)]. Os procedimentos foram conduzidos por um pesquisador, cego quanto aos grupos e que desconhecia os tratamentos.

**Figura 1:**
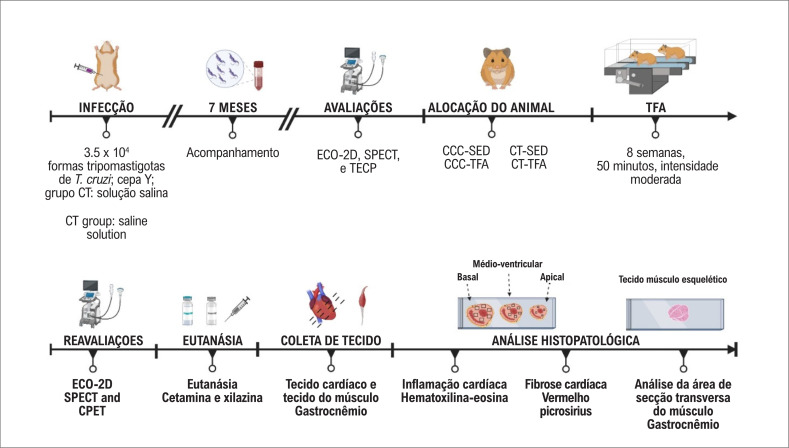
Linha do tempo do estudo. Após infecção experimental com cepa Y de T. cruzi e sete meses de acompanhamento para o desenvolvimento da doença, os animais sobreviventes e o grupo controle foram submetidos à ecocardiografia bidimensional (ECO-2D), tomografia computadorizada por emissão de fóton único (SPECT) com ^99m^Tc-Sestamibi e Teste de Esforço Cardiopulmonar (TECP). Em seguida, os animais foram alocados em quatro grupos [CT-SED (n=6), CCC-SED (n=16), CT-TFA (n=8), CCC-TFA (n=12)]. Subsequentemente, os grupos TFA foram submetidos a oito semanas de Treinamento Físico Aeróbico (TFA) e reavaliados. Por fim, os animais foram eutanasiados, e o coração e o músculo esquelético foram coletados para análise histopatológica. A figura foi criada por meio do BioRender.com

Os animais foram infectados por via intraperitoneal com 3,5x10^4^ da forma tripomastigota da cepa Y de *T. cruzi*, enquanto o grupo controle foi inoculado com solução salina (0,4 mL). Sete meses após a infecção (Figura 1), os animais foram submetidos à ecocardiografia bidimensional (ECO-2D), tomografia computadorizada por emissão de fóton único (SPECT) com ^99m^Tc-Sestamibi (RPHKARDIA, Porto Alegre, Brasil) e Teste de Esforço Cardiopulmonar (TECP). As avaliações das imagens duraram uma semana, com um intervalo de dois a três dias entre os testes. O TECP durou cinco dias a mais para climatização e realização dos testes. O treinamento físico foi iniciado dois dias depois do TECP. Oito semanas após o período de intervenção (TFA), os animais foram submetidos às mesmas avaliações (TECP, ECO-2D e SPECT), em seguida à eutanásia [cetamina (Vetbrands, Jacareí, São Paulo, Brasil) e xilazina (Bayer, São Paulo, Brasil), 160 mg/Kg e 10 mg/Kg, respectivamente], seguida de coleta de amostras de sangue para confirmar a doença de Chagas crônica.^48^ A infecção crônica por *T. cruzi* foi confirmada pela técnica de *Western blotting* para detectar anticorpos anti-*T.cruzi* no soro de animais infectados conforme descrito anteriormente.^48,49^ Amostras do tecido cardíaco e do músculo esquelético foram coletados para análise histopatológica.

O estudo foi realizado seguindo-se as recomendações do CONCEA após aprovação do Comitê de Ética no Uso de Animais – CEUA (N°229/2019) de nossa instituição.

### Ecocardiografia bidimensional

O ecocardiograma com Doppler foi realizado usando um sistema de ecocardiografia bidimensional de alta resolução (ECO-2D) para pequenos animais Vevo® 2100 (*Visual Sonics Inc*., Toronto, Canada) com um transdutor linear com frequência de 30 MHz. Após sedação com cetamina e xilazina (80 mg/Kg e 5 mg/Kg, respectivamente), os pelos dos animais foram raspados e os animais colocados em decúbito lateral. Foram obtidas imagens da janela paraesternal (eixo curto e longo) do Ventrículo Esquerdo (VE). Imagens bidimensionais foram usadas para avaliar FEVE, volume diastólico final do VE e volume sistólico final do VE no corte paraesternal eixo longo como descrito anteriormente.^50^

### Imagem de perfusão miocárdica

Para avaliar a perfusão miocárdica em repouso, foram adquiridas imagens por SPECT com ^99m^Tc-Sestamibi, usando uma gamma camera (BrightView XCT; Philips Medical Systems Inc., Cleveland, OH) adaptada com um sistema de aquisição de imagens e um colimador tipo "*pinhole*" de abertura de 1,5mm posicionado paralelamente a um suporte rotacional para o animal, conforme descrito anteriormente.^48,51^

Em resumo, sob anestesia com isoflurano^52^ (Isoforine, São Paulo, Brasil), os animais receberam 555 MBq de MIBI pela veia sublingual e acordaram. Noventa minutos depois, os hamsters foram anestesiados novamente com uma combinação de cetamina (80mg/Kg) e xilazina (5mg/kg), e imagens SPECT foram adquiridas. As imagens, coletadas com os animais em pé, foram reconstruídas usando um algoritmo tridimensional de subconjuntos ordenados e maximização da expectativa (3D-OSEM, quatro subconjuntos e 10 interações). O acúmulo de radiotraçador no miocárdio foi analisado de maneira semiquantitativa utilizando mapas polares gerados pelo software MunichHeart® (MunichHeart software, Technical University Munich, Munique, Alemanha). Os DPM foram considerados significativos se superiores a 5% do VE.^48^

### Teste de esforço cardiopulmonar

Antes do TECP, os animais foram familiarizados com a esteira por cinco dias consecutivos, com incremento de velocidade e inclinação fixa (5°).^53^ Para a avaliação da capacidade cardiorrespiratória dos animais, o consumo de oxigênio (VO_2_) e a produção de dióxido de carbono (VCO_2_) foram continuamente medidos por calorimetria indireta de fluxo aberto (Panlab, Harvard Apparatus, Espanha). O VO_2_ foi expresso em unidades ajustadas para o tamanho do animal (mL.kg^-0.75^.min^-1^) e o pico de VO_2_ foi definido como o valor mais alto de VO_2_ medido durante o teste antes da exaustão. O VO_2_ no limiar anaeróbico (VO_2LA_) foi definido como o consumo de oxigênio no qual uma relação linear entre VCO_2_ e VO_2_ foi perdida durante exercício progressivo na esteira, associado a um aumento abrupto na razão de troca respiratória.

O protocolo de estresse foi descrito anteriormente.^54^ Em resumo, o protocolo consiste em aumentar a velocidade e a inclinação da esteira em cada estágio. Os primeiros três estágios tiveram duração de dois minutos com um aumento de cinco graus em cada estágio. Do estágio quatro em seguida, a duração foi de um minuto, e a inclinação mantida em 15 graus. A velocidade iniciou-se em 15 cm/s e aumentada 5 cm/s até o estágio seis. A partir daí, a velocidade aumentou 1,67 cm/s em cada estágio até a exaustão do animal. Os critérios para interrupção do teste foram o animal permanecer por cinco segundos ou mais sobre a grade de estimulação elétrica ou permanecer por 10 segundos sobre a extremidade final da esteira.^53,54^

### Treinamento físico aeróbico

Após a avaliação basal (ECO-2D, SPECT e TECP), os animais foram submetidos ao TFA em uma esteira (Gaustec Magnetismo, Nova Lima, Minas Gerais, Brasil), seguindo um protocolo de oito semanas adaptado de um estudo anterior.^53^ O TFA foi realizado cinco vezes por semana durante 50 minutos em intensidade moderada (50% da velocidade pico definida pelo TECP e inclinação de 5%) no mesmo período do dia. O tempo, a velocidade e o grau foram aumentados progressivamente nas duas primeiras semanas até se atingir a intensidade prescrita. Para assegurar uma manipulação e uma exposição ao TFA similares aos animais, os animais sedentários foram submetidos a dois minutos de corrida na esteira cinco dias por semana, com a mesma velocidade de corrida que os animais submetidos ao TFA. Após o período de treinamento, os métodos de avaliação foram repetidos e os animais submetidos à eutanásia para coleta de tecidos e análise histopatológica.

### Análise histopatológica

Para a análise histopatológica, secções transversais (5 μm espessura) foram obtidas de três regiões do coração (basal, médio-ventricular e apical), mantendo a orientação para a correlação com imagens *in vivo* como descrito anteriormente.^10^ Após progressiva desidratação, o tecido foi fixado em parafina e amostras de cada seção ventricular coradas com hematoxilina-eosina e picrosirius vermelho para quantificar inflamação e fibrose, respectivamente. Em seguida, imagens digitais microscópicas (lentes objetivas 40x) do endocárdio, miocárdio e epicárdio de cada segmento de VE foram obtidas usando o microscópio BX51 Olympus (Olympus; Tóquio, Japão), equipado com uma câmera Q-color 5 (Olympus America, Center Valley, Inc., EUA). Para os cortes corados com picrosirius vermelho, também foram tiradas fotos por microscopia de luz polarizada para quantificar colágeno tipo I e tipo III, identificando as fibras vermelho-amarelas e verdes, respectivamente. Em seguida, as fotos foram analisadas usando os programas Aperio ImageScope (versão 12.4.6, Leica Biosystems Imaging, Inc., EUA) e Image Pro Plus 32 (versão 4.5.0.29; Media Cybernetics, Inc., Maryland, EUA).

A inflamação foi quantificada contando-se o número de células mononucleadas por campo (células/mm^2^). A coloração com picrosirius vermelho definiu fibrose intersticial como porcentagem (%) da área total com cuidado para excluir fibrose perivascular. Para analisar as alterações histopatológicas, o VE foi dividido em 16 segmentos: basal (anterior, ânterosseptal, ínferosseptal, inferior, ínfero-lateral, ântero-lateral), médio-ventricular (anterior, ânterosseptal, ínferosseptal, inferior, ínfero-lateral, ântero-lateral) e apical (anterior, septal, inferior, lateral).

Cortes transversais do músculo esquelético (5 μm) da porção medial foram fixadas em parafina e, em seguida, coradas com hematoxilina-eosina para quantificar a AST. Imagens histológicas da porção medial do músculo (escala=100μm, 20x) foram analisadas usando os programas Aperio ImageScope (versão 12.4.6, Leica Biosystems Imaging, Inc., EUA) e ImageJ Fiji (versão JAVA 1.8.0_322., National Institutes of Health, Bethesda, Maryland, EUA). Para a análise da AST, cerca de 200 fibras musculares foram medidas por amostra. Além disso, usamos somente um número representativo de animais [CT-SED (n=4), (C) CCC-SED (n=6), (D) CT-TFA (n=4) e (E) CCC-TFA (n=5)] de cada grupo experimental.

### Cálculo do tamanho amostral

O tamanho da amostra foi calculado usando o programa *online* OpenEpi (Open Source Epidemiologic Statistics for Public Health, version 3.1, Atlanta, GA, EUA), e os critérios usados para definir o tamanho amostral foram baseados em estudos prévios.^10,55^ Assumiu-se uma redução de 10% no defeito de perfusão entre os grupos infectados ao final do tratamento, com um alfa bicaudal de 0,05, 1-β= 0.8. O tamanho amostral estimado para este estudo foi 13 animais em cada grupo infectado e oito animais nos grupos controles. No entanto, considerou-se uma perda de 40% dada a agressividade da parasitemia nos grupos de animais infectados. Também consideramos que somente 30-50% dos animais infectados cronicamente desenvolvem cardiomiopatia chagásica.^12^ Assim, o número total de animais usados neste estudo foi 16 animais controles (CT-SED = 8 animais; CT-TFA = 8 animais) e 44 animais infectados (CCC sedentários = 22 animais; CCC-TFA = 22 animais), totalizando 60 animais.

### Análise estatística

As variáveis contínuas foram descritas como média ± desvio padrão, e as variáveis categóricas em frequência absoluta (n) e relativa (%). O teste de Kolmogorov-Smirnov foi usado para verificar distribuição gaussiana das variáveis. Análise de variância *one-way* foi usada para comparação simultânea dos quatro grupos experimentais no basal e para análise histopatológica da inflamação e fibrose. A análise de variância mista (*mixed* ANOVA ou ANOVA fatorial *split-plot*) para medidas repetidas foi usada para verificar a interação (efeito principal) entre os grupos experimentais (efeito entre indivíduos) e o tempo (efeito intraindivíduo) para o efeito do TFA sobre as variáveis da ECO-2D, do SPECT e do TECP. Em caso de interações estatisticamente significativas, múltiplas comparações post hoc de Bonferroni foram realizadas.

A análise estatística e os gráficos foram elaborados usando o programa GraphPad Prism (versão 9.0.0; GraphPad Software, San Diego, Califórnia, EUA). O nível de significância foi de 5% em todas as análises (p<0.05).

## Resultados

Na fase aguda da infecção (até 35 dias após a infecção), observou-se uma taxa de mortalidade de 16% (n=7). Trinta e sete animais infectados e 16 controles foram submetidos a avaliações basais (sete meses após a infecção). Durante as avaliações basais, três animais infectados morreram devido a anestesia. Seis animais infectados (CCC-SED, n= 4 e CCC-TFA, n= 2) e dois controles morreram durante o período de intervenção. Nenhum animal morreu durante o TFA.

### TFA reduz remodelamento, e disfunção sistólica e de perfusão do VE

Os resultados do ECO-2D e do SPECT de perfusão do miocárdio no basal e após as avaliações após o TFA estão apresentados na Tabela 1. Em relação à FEVE, observou-se uma interação significativa entre o tempo e os grupos experimentais. A FEVE mostrou uma importante redução somente no grupo CCC-SED. O grupo CCC-TFA foi o único que apresentou dilatação diastólica do VE ao longo do tempo. No entanto, o grupo não apresentou aumento na massa ou no diâmetro sistólico do VE. Em relação aos DPM, o grupo CCC-SED apresentou piora dos defeitos ao longo do tempo.

**Tabela 1:** Dados de ecocardiografia e de tomografia computadorizada por emissão de fóton único (SPECT) dos grupos experimentais antes e após o treinamento físico aeróbico

Variáveis	CT-SED (n=6)	CCC-SED (n=16)	CT-TFA (n=8)	CCC-TFA (n=12)	Valor p
**FEVE (%)**					
Antes	52,93 ± 1,49	45,49 ± 8,84	49,86 ± 2,93	43,74 ± 9,92	
Após	52,47 ± 3,59†	39,69 ± 7,80*	47,28 ± 5,67†	42,46 ± 4,47	0,007
**DDVE (mm)**					
Antes	7,71 ± 0,51	7,57 ± 0,70	7,73 ± 0,34	7,71 ± 0,43	
Após	7,96 ± 0,56	7,99 ± 1,00	8,22 ± 0,50	8,34 ± 0,60*	0,030
**DSVE (mm)**					
Antes	5,68 ± 0,43	5,68 ± 0,94	5,99 ± 0,40	6,13 ± 0,54	
Após	6,08 ± 0,53	6,06 ± 1,01	6,47 ± 0,81	6,80 ± 0,74	0,050
**Massa do VE (mg)**					
Antes	519,19 ± 89,55	549,85 ± 96,73	532,02 ± 98,15	541,89 ± 84,71	
Após	667,78 ± 125,04*	686,99 ± 152,06*	735,47 ± 170,16*	622,15 ± 129,83	0,014
**DPM (%)**					
Antes	2,45 ± 1,69	4,64 ± 3,45	3,08 ± 2,56	4,81 ± 3,52	
Após	3,31 ± 2,44†	8,29 ± 7,06*	2,56 ± 3,43	6,30 ± 3,38	0,010

Dados em média e desvio padrão; TFA: treinamento físico aeróbico, CCC: cardiomiopatia chagásica crônica, CT: controle, SED: sedentário, FEVE: fração de ejeção do ventrículo esquerdo; DDVE: diâmetro diastólico do ventrículo esquerdo, DSVE: diâmetro sistólico do ventrículo esquerdo, VE: ventrículo esquerdo, DPM: defeito de perfusão do miocárdio; ANOVA mista para medidas repetidas;

Os animais que apresentavam DPM importantes (>5% do VE) foram detectados nos dois grupos de animais no basal, com cinco animais (36%) no grupo sedentário e três (25%) no grupo exercício. Após o seguimento, observamos um aumento no tamanho (p<0,05) e no número de animais (n=10, 67%) com DPM no grupo CCC-SED, enquanto no grupo submetido ao TFA observamos um menor aumento no tamanho (p>0,05) e no número de animais com anormalidades de perfusão (n= 5, 42%).

**Figura 2:**
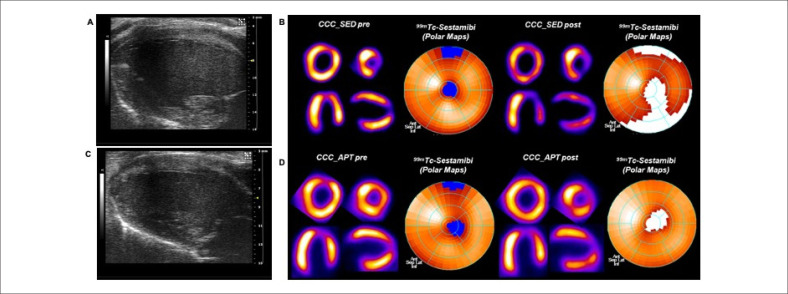
Efeitos do Treinamento Físico Aeróbico (TFA) na função e na perfusão cardíaca; (A) e (B) mostram imagens representativas da ecocardiografia bidimensional (ECO-2D), tomografia computadorizada por emissão de fóton único (SPECT) com Defeitos de Perfusão Miocárdica (DPM) de um animal do grupo CCC-SED (n=16), e (C) e (D) de um animal do grupo CCC-TFA (n=12); CCC: cardiomiopatia chagásica crônica, SED: sedentário

A Figura 2 mostra um exemplo de um animal do grupo CCC-SED que apresentou dilatação do Diâmetro Sistólico do VE (DSVE) associada com aumento de DPM ao longo do tempo (Figuras 2 A e B) em comparação a um animal do grupo CCC-TFA com função e morfologia do VE preservadas e sem aumento nos DPM (Figuras 2 C e D).

### TFA aumenta a eficiência da aptidão cardiorrespiratória na CCC

As variáveis do TECP estão na Tabela 2. Além de os animais não terem apresentado melhoras significativas no consumo de oxigênio no pico de exercício ou no limiar anaeróbico, o grupo CT-SED foi o único que não apresentou aumento no tempo até a exaustão após o período de acompanhamento. No entanto, os grupos que se submeteram ao treinamento físico apresentaram melhoras mais evidentes. Embora o CCC-TFA tenha apresentado um aumento no VO_2AT_, e uma diminuição tenha sido observada no CCC-SED, essa diferença não alcançou significância estatística..

**Tabela 2:** Dados de Teste de Esforço Cardiopulmonar dos grupos experimentais antes e após o treinamento físico aeróbico

Variáveis	CT-SED (n=6)	CCC-SED (n=16)	CT-TFA (n=8)	CCC-TFA (n=12)	Valor p
**Tempo até exaustão (s)**					
Antes	464,67 ± 32,47	445,21 ± 72,19	494,00 ± 71,25	481,20 ± 76,57	
Após	557,50 ± 91,24	539,64 ± 94,04*	686,78 ± 109,59*†	687,20 ± 141,67*†	0,043
**VO**_**2 pico**_ **(mL.Kg.min)**					
Antes	38,50 ± 3,46	42,70 ± 5,45	42,11 ± 4,40	40,32 ± 5,08	
Após	43,04 ± 4,90	43,75 ± 4,42	43,33 ± 3,43	45,60 ± 5,65*	0,254
**VO**_**2@LA**_ **(mL.Kg.min)**					
Antes	31,22 ± 6,79	33,06 ± 5,48	30,12 ± 4,54	30,56 ± 3,25	
Após	30,90 ± 6,25	28,95 ± 5,86	33,44 ± 3,96	34,39 ± 4,06†	0,759

Dados em média e desvio padrão; TFA: treinamento físico aeróbico, CCC: cardiomiopatia chagásica crônica, CT: controle, SED: sedentário, VO_2_: consumo de oxigênio; VO_2AT_: consumo de oxigênio no limiar anaeróbico; ANOVA mista para medidas repetidas;

* p<0,05 vs. basal do mesmo grupo experimental,

† p≤0,05 vs. CCC-SED após tratamento, teste de Bonferroni para comparações múltiplas.

### TFA reduz inflamação e fibrose no miocárdio e preserva a AST do músculo esquelético

**Figura 3:**
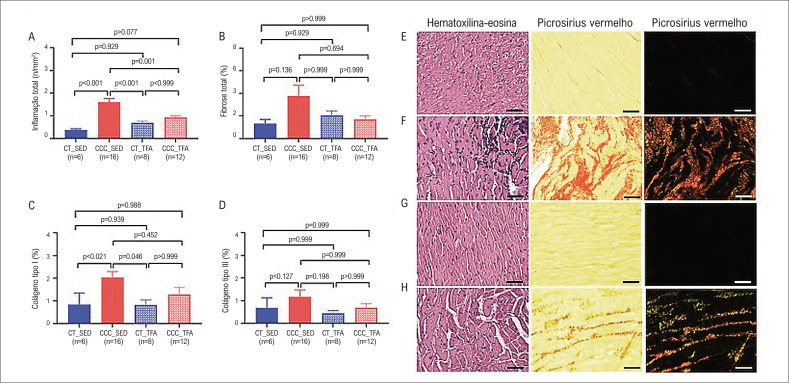
Gráficos de barra mostrando os resultados de (A) inflamação miocárdica, (B) fibrose total, (C) colágeno tipo I, e (D) colágeno tipo III. Amostras histopatológicas representativas de tecidos de animais dos grupos (E) CT-SED (n=6), (F) CCC-SED (n=16), (G) CT-TFA (n=8) e (H) CCC-TFA (n=12), marcados com hematoxilina-eosina e vermelho picrosirius; TFA: Treinamento Físico Aeróbico; CCC: Cardiomiopatia Chagásica Crônica, CT: Controle, SED: Sedentário; escala= 50 μm, aumento de 40x.

A inflamação do miocárdio foi maior nos animais do grupo CCC-SED em comparação aos grupos CT-SED, CT-TFA e CCC-TFA (1,61±0,63 vs. 0,37±0,12 vs. 0,7±0,2 vs. 0,93±0,2 células/mm^2^, respectivamente, p<0,001). Não foi observada diferença significativa na fibrose total (p>0,05). Contudo, o grupo CCC-SED, mas não o grupo CCC-APT, apresentou maior expressão de colágeno tipo I em comparação aos grupos controles (p<0,05) (Figura 3).

**Figura 4:**
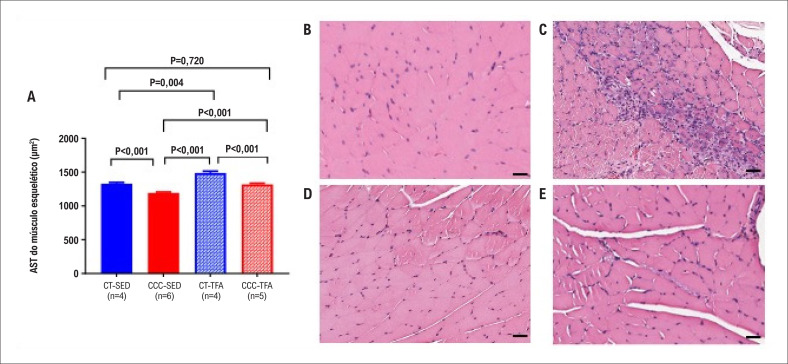
(A) Gráficos de barra mostrando a análise quantitativa da área de secção transversa do músculo gastrocnêmio de animais do grupo infectado e do grupo controle. Imagens histopatológicas representativas do músculo gastrocnêmio dos grupos (B) CT-SED (n=4), (C) CCC-SED (n=6), (D) CT-TFA (n=4) e (E) CCC-TFA (n=5). Infiltrado inflamatório difuso de células mononucleares pode ser visto em CCC-SED (C); TFA: Treinamento Físico Aeróbico; CCC: Cardiomiopatia Chagásica Crônica; CT: Controle; SED: Sedentário; escala =100 μm, aumento 20x.

Além disso, a atrofia do músculo esquelético foi confirmada pela redução na AST. O CCC-SED apresentou atrofia do músculo esquelético que foi normalizada pelo treinamento físico no grupo CCC-APT, como observado na Figura 4. Os animais do grupo CCC-SED frequentemente apresentaram inflamação do músculo esquelético que foi menos observado e menos intenso nos animis CCC-TFA.

## Discussão

O presente estudo investigou os efeitos do TFA em um modelo experimental de CCC em hamsters sírios utilizando técnicas de imagens de alta resolução *in vivo* e TECP. Os principais achados deste estudo foram que o TFA atenuou a progressão dos DPM, remodelamento e deterioração sistólica do VE, além de melhorar a eficiência da ACR. Ademais, o TFA diminuiu a inflamação e fibrose do miocárdio e aumentou a AST do músculo esquelético.

Em nosso estudo, o grupo CCC-SED apresentou piora nos defeitos de perfusão ao longo do tempo. A piora progressiva nos DPM como um mecanismo fisiopatológico da doença foi observada previamente tanto em cenários experimentais^48,55^ como clínicos.^56,57^ Além disso, alguns estudos levantaram a hipótese de que anormalidades da perfusão miocárdica podem preceder a deterioração sistólica do VE.^19,58^ Portanto, terapias visando melhorar a perfusão do miocárdio podem prevenir a progressão no dano cardíaco. Em nosso estudo, não observamos melhora nos DPM após a intervenção com exercício; contudo, os defeitos de perfusão não aumentaram significativamente após o exercício, diferentemente do que foi observado no grupo sedentário infectado.

Com base em nosso conhecimento atual, este é o primeiro estudo a utilizar esta estratégia para tratar DPM na CCC. Um recente estudo piloto investigou os efeitos do exercício aeróbico sobre os DPM em pacientes com angina microvascular, uma doença com um mecanismo similar aos distúrbios de perfusão miocárdica.^59^ Os autores observaram uma redução significativa nos DPM associados com melhora no pico de VO_2_ e na qualidade de vida. O exercício também promoveu benefícios na perfusão miocárdica na doença arterial coronariana^60,61^ e insuficiência cardíaca.^62,63^ Os mecanismos pelos quais o treinamento com exercício melhora a perfusão cardíaca são provavelmente multifatoriais e vários foram descritos incluindo melhora na função endotelial,^64–66^ adaptações vasculares coronarianas,^67^ e melhor colateralização.^68^ Além disso, a redução da inflamação e melhorias no equilíbrio autonômico e neuro-hormonal podem contribuir para isso.

Quanto a outros tratamentos para perfusão miocárdica na CCC, Tanaka et al.^55^ utilizaram dipiridamol, um agente vasodilatador da artéria coronária, para melhorar DPM em hamsters sírios fêmeas. Os autores observaram uma melhora significativa nos DPM nos grupos tratados em comparação aos grupos controles. No entanto, o tratamento não interrompeu a disfunção progressiva do VE. Segundo os autores, apesar da melhora na perfusão, a doença progrediu porque o tratamento não teve benefícios na inflamação do miocárdio, que permaneceu similar entre os dois grupos infectados. Recentemente, Tanaka et al.^17^ investigaram o mesmo modelo animal com CCC tratada com pentoxifilina. Os autores relataram que esse tratamento reduziu a inflamação e os DPM, mas não preveniu a progressão da disfunção sistólica do VE. Os autores sugeriram que talvez o período de intervenção (seis meses após a infecção) não tenha sido suficiente para melhorar a disfunção sistólica do VE, mas para reduzir inflamação e DPM, uma vez que esses ocorrem nos estágios iniciais da doença e precedem a disfunção sistólica do VE. Outro estudo, utilizando verapamil (bloqueador de canal de cálcio) e aspirina (anti-inflamatório não esteroidal), demonstrou melhora significativa nos DPM e na qualidade de vida de pacientes com CCC.^69^ Infelizmente, os autores não avaliaram a função sistólica nem inflamação após a intervenção. Os efeitos benéficos do verapamil na CCC também foram demonstrados em camundongos infectados com *T. cruzi*.^9,70^

A infecção crônica pelo *T. cruzi* em humanos geralmente leva a um dano cardiovascular mais agressivo, com maior quantidade de fibrose e remodelamento ventricular mais grave em homens que em mulheres.^71^ Em animais, o pico de parasitemia e a progressão da doença parecem mais homogêneos nas mulheres.^55,72^ Uma vez que vários estudos^10,17,21,43,48,55^ apresentaram, com sucesso, o uso de hamsters sírios fêmeas na investigação da CCC, escolhemos esse modelo murino para estudar a doença. Nossos resultados estão de acordo com pesquisas prévias 10,43 em que hamsters sírios desenvolveram a doença semelhante a CCC em humanos. Disfunção ventricular esquerda, um marcador de gravidade da doença, foi observada na primeira avaliação sete meses após a infecção parasitária, quando a FEVE já estava reduzida. Achados similares foram descritos por Ribeiro et al.^73^ em que a FEVE e o diâmetro sistólico final do VE estavam deteriorados aos seis meses do início da doença, com progressão em estágios mais tardios nesse mesmo modelo animal. No entanto, observamos que o exercício interrompeu a progressão da disfunção do VE. Poucos ensaios clínicos investigaram a função e a morfologia do VE após o exercício.^31,74^ Nenhum desses estudos documentaram melhoras na função ou na morfologia cardíaca após o exercício. Entretanto, trataram-se de estudos pequenos, com diferentes perfis de pacientes e, portanto, mais estudos com um maior número de pacientes e períodos mais longos de acompanhamento são necessários para investigar se o treinamento físico pode melhorar ou interromper a deterioração cardíaca na CCC.

Além disso, na análise histopatológica, observamos que os animais infectados sedentários mostraram uma maior extensão de infiltrados inflamatórios com agrupamentos de células inflamatórias mononucleares e áreas de fibrose extensa em comparação a outros animais. O papel da inflamação e da fibrose promovendo anormalidades de perfusão já foi relatado por outros pesquisadores. Bilate et al.^43^ encontraram uma correlação estatisticamente significativa entre miocardite e fibrose intersticial. Os autores sugeriram que, provavelmente, o infiltrado inflamatório cardíaco tenha sido responsável por essa lesão tecidual progressiva, e consequente remodelamento e fibrose extensa. Ademais, Oliveira et al.^48^ relataram que esses distúrbios de perfusão possam estar localizados em regiões com miocárdio viável e perfusão diminuída secundária à inflamação. Assim, demonstramos que o TFA realizado em intensidade moderada, cinco vezes por semana, por oito semanas, foi capaz de reduzir inflamação e fibrose. A inflamação do miocárdio é a principal característica histopatológica da CCC, e terapias que objetivam melhorar essa condição demonstraram benefício promissores na função cardíaca.^1,75^

A fibrose cardíaca é considerada um preditor independente de desfecho adverso nesta cardiomiopatia.^76^ A fibrose também exerce um importante papel no déficit do desempenho cardíaco e dilatação cardíaca.^77^ Ramirez et al.^21^ relataram dilatação cardíaca (principalmente no apex) e trombo mural em hamsters sírios com CCC. Em nosso estudo, observamos dilatação diastólica no grupo CCC-TFA ao longo do tempo. No entanto, nossa hipótese é a de que esse aumento tenha sido uma adaptação fisiológica ao exercício, uma vez que nenhuma diferença foi observada na FEVE ou na fibrose entre esse grupo e animais não infectados. O colágeno tipo I foi mais abundante no grupo CCC-SED, mas não identificamos nenhuma diferença estatisticamente significativa entre os grupos com doença de Chagas que se submeteram ao exercício.

Os pacientes com CCC também podem apresentar anormalidades no músculo esquelético.^20^ Em nosso estudo, nós analisamos amostras do músculo gastrocnêmio. O grupo CCC-SED apresentou atrofia, e no grupo CCC-TFA a atrofia muscular foi normalizada após o exercício. Embora alguns estudos prévios^20–25,78–81^ tenham avaliado anormalidades do músculo esquelético após a infecção por *T. cruzi*, somente alguns deles analisaram a fase crônica, e nenhum avaliou AST do músculo após o TFA. Em relação aos estudos experimentais, Silva et al.^80^ observaram miosite com exsudato mononuclear e fibrose nos músculos do diafragma, intercostal e psoas de coelhos seis meses após a infecção.^80^ Weaver et al.^22^ avaliaram o músculo quadríceps de camundongos no início (2-4 meses) e no final (9-10 meses) da fase crônica. Os autores relataram poucos parasitas *T. cruzi* no músculo, inflamação, vasculite necrotizante, fibrose vascular, fibrose do endomísio, e anormalidades na marcha (incluindo evitar colocar o peso sobre um dos membros, arrastar o pé, e até mesmo paresia).^22^ Ramírez et al.^21^ avaliaram o músculo esquelético (músculo não especificado) de hamsters sírios e relataram miosite focal e necrose. Souza et al.^78^ identificaram uma expressão aumentada de quimiocinas, e que o número de células inflamatórias mantiveram-se elevadas no músculo esquelético (músculo não especificado) de camundongos em todos os tempos avaliados entre a fase aguda e crônica. Estudos sobre CCC com análises de biópsias humanas relataram dano capilar e menor AST do músculo esquelético (músculo vasto lateral);^23^ inflamação, desorganização e atrofia das fibras musculares (músculo bíceps);^20^ fibras musculares atróficas desnervadas (músculo gastrocnêmio);^24^ oclusão capilar, maior percentual de fibras musculares com menor capacidade oxidativa e maior percentual de fibras com maior capacidade glicolítica (músculo vasto lateral).^25^

Independentemente dos benefícios bem estabelecidos do exercício aeróbico em pacientes cardiopatas,^45,82^ poucos estudos clínicos investigaram o TFA no tratamento de cardiomiopatia chagásica.^30^ Lima et al.^32^ observaram melhoras na aptidão cardiorrespiratória (aumento no pico de VO_2_, no tempo de exercício e da distância de caminhada no Teste de Caminhada de Seis Minutos) e nenhum efeito adverso do exercício nos pacientes tratados. Artigos do estudo PEACH^29,31,35,83^ abordaram os efeitos do treinamento com exercício em pacientes com cardiomiopatia chagásica. Os estudos observaram maiores benefícios na ACR em pacientes com disfunção ventricular esquerda e insuficiência cardíaca. Adicionalmente, os efeitos benéficos contínuos do exercício ainda eram notados aos três48 e seis31 meses de acompanhamento. O exercício realizado por seis meses também melhorou a resposta vascular cutânea à hiperemia reativa.^35^

Nosso estudo também detectou efeitos positivos do TFA na CCC, com aumento na capacidade de exercício nos grupos submetidos ao TFA após oito semanas de treinamento (intensidade moderada, cinco dias/semana, 50 minutos). Quanto aos estudos experimentais prévios, somente seis^36–38,84–86^ investigaram os efeitos do TFA em outras fases da doença de Chagas, e somente um^39^ abordou o papel do exercício aeróbico na CCC já estabelecida. Schebeleski-Soares et al.^86^ conduziram um treinamento físico na esteira durante oito semanas (intensidade moderada, cinco dias/semana, velocidade e duração progressivas) antes da infecção por *T. cruzi* em camundongos. Soares et al.^37^ realizaram um treino na esteira por oito semanas (intensidade moderada, cinco dias/semana, aumento progressivo de velocidade e duração) antes da infecção por *T. cruzi* em camundongos. Novaes et a.^85^ aplicaram um protocolo de esteira de nove semanas (intensidade moderada, cinco dias/semana, aumento progressivo de velocidade e duração) em ratos Wistar antes da infecção por *T. cruzi*. Novaes et al.^84^ também usaram o mesmo protocolo em outra investigação com ratos Wistar (nove semanas de exercício na esteira, intensidade moderada, cinco dias/semana, aumento progressivo de velocidade e duração) antes da infecção por *T. cruzi*. Lucchetti et al.^36^ conduziram um protocolo na esteira durante nove semanas (intensidade definida pelo máximo estado estável de lactato, cinco dias/semana, 60 minutos)^87^ antes da infecção por *T. cruzi* em camundongos. Pedra-Rezende et al.^38^ estudaram a forma indeterminada crônica da doença de Chagas e usaram um protocolo de quatro semanas na esteira 140 dias após infecção (intensidade moderada, cinco dias/semana, aumento progressivo de velocidade e 60 minutos de duração). Finalmente, Preto et al.^39^ usaram um protocolo de natação por oito semanas (baixa intensidade de exercício aeróbico, cinco dias por semana, 30 minutos por dia) para tratar camundongos com CCC.

Embora todos esses estudos tenham usado o exercício aeróbico para tratar os animais, atualmente, somente Preto et al.^39^ observaram os efeitos do TFA na CCC e mostraram que o exercício melhorou parâmetros morfológicos e morfométricos dos ventrículos esquerdo e direito. Já nos animais não tratados, os autores observaram déficit na função contrátil do cardiomiócitos, mais inflamação e quantidades maiores de colágeno, hipertrofia do VE, diminuição na área periférica dos cardiomiócitos, alterações microvasculares e piora da tolerância ao exercício.^39^ Considerando esse cenário, nossos resultados contribuem com novos conhecimentos sobre os efeitos do exercício aeróbico na perfusão miocárdica e alterações histopatológicas no coração, e mostram que o TFA foi capaz de preservar a AST do músculo esquelético. Nós destacamos que este foi o primeiro estudo experimental em hamsters sírios que usou o exercício aeróbico no tratamento da CCC com o objetivo de mitigar os DPM, a inflamação e a fibrose no tecido cardíaco. Este também foi o primeiro estudo a avaliar a integridade da área periférica do músculo esquelético de animais com CCC após oito semanas de exercício aeróbico.

Como uma limitação, nós estudamos somente defeitos de perfusão no repouso, e é esperado que defeitos isquêmicos de perfusão ajudariam na interpretação. No entanto, seria necessário usar agentes inotrópicos positivos em animais anestesiados, o que pode interferir nos resultados. Adicionalmente, nosso grupo mostrou anteriormente a correlação entre DPM e inflamação em miocárdio viável.^48,55^ Apesar dos resultados positivos sobre os efeitos do TFA, talvez um período mais longo de acompanhamento e de treinamento com exercício revelaria resultados mais significativos sobre DPM. Além disso, nós não incluímos neste artigo dados quantitativos da inflamação no músculo esquelético. Estudos futuros usando treinamento com exercício no manejo de CCC são necessários para contribuir ao conhecimento disponível.

Por fim, este estudo corrobora a hipótese de que tanto DPM como inflamação contribuem para a deterioração na função sistólica na CCC, e o exercício é uma importante estratégia para minimizá-los. O treino com exercício tornou-se uma forte recomendação para a maioria das doenças cardíacas. Contudo, diretrizes atuais não recomendam explicitamente reabilitação cardíaca para pacientes com CCC.^1,88^ Nossos resultados fornecem a base para estudos futuros que visem investigar os benefícios da intervenção com exercício na CCC.

## Conclusão

Nosso estudo apresenta evidências de que o TFA minimiza a disfunção cardíaca e os DPM em um modelo de hamster sírio com CCC. Ademais, além de melhorar o desempenho na corrida, o APT reduziu a infiltração de células inflamatórias e a fibrose no miocárdio, indicando seu potencial como estratégia terapêutica para a CCC. Esses achados destacam a importância do treinamento com exercício em mitigar a progressão da CCC e melhorar a função cardíaca. Além dos achados significativos relacionados às alterações cardíacas, vale destacar que nosso estudo também observou atrofia musculoesquelética em hamsters com CCC e melhora na AST do músculo após treinamento físico. Embora essas alterações não tenham sido o foco de nosso estudo, elas forneceram outros *insights* sobre complicações sistêmicas associadas a essa doença. Portanto, um entendimento mais abrangente dos efeitos do TFA sobre várias dimensões fisiopatológicas da CCC continua sendo um aspecto importante para estudos futuros.
