# Supplementary material for: Treinamento Físico Reduz a Inflamação e a Fibrose e Preserva a Função e a Perfusão Miocárdica em um Modelo de Cardiomiopatia Chagásica Crônica
Source: Arq Bras Cardiol. 2024 Aug 21;121(8):e20230707. [Article in Portuguese] doi: 10.36660/abc.20230707 (PMC11495816; doi:10.36660/abc.20230707)
Supplement: Translation

# Translation: Exercise Training Reduces Inflammation and Fibrosis and Preserves Myocardial Function and Perfusion in a Model of Chronic Chagas Cardiomyopathy

**DOI:** 10.36660/abc.20230707i

**Authors:** Thayrine R. Damasceno, Denise M. Tanaka, Enrico F. Magnani, Rafael D. B. Oliveira, Danielle A. G. Pereira, Ildernandes Vieira-Alves, Virginia S. Lemos, Jorge M. Cabeza, Camila G. Fabricio, Alessandra A. Resende, Dawit A. P. Gonçalves, Gustavo de Oliveira Zanetti, Eduardo E. Vieira de Carvalho, Marcus V. Simões, Luciano F. L. Oliveira

**Affiliations:** 1 Belo Horizonte MG Brazil Universidade Federal de Minas Gerais, Belo Horizonte, MG – Brazil; 2 Ribeirão Preto SP Brazil Faculdade de Medicina de Ribeirão Preto – Universidade de São Paulo, Ribeirão Preto, SP – Brazil; 3 São Paulo SP Brazil Hospital Israelita Albert Einstein, São Paulo, SP – Brazil; 4 Uberaba MG Brazil Universidade Federal do Triângulo Mineiro, Uberaba, MG – Brazil

**Keywords:** Chagas Cardiomyopathy, Myocarditis, Cardiorespiratory Fitness, Exercise, Myocardial Perfusion Imaging

## Abstract

**Background:**

Chronic Chagas cardiomyopathy (CCC) is caused by an inflammatory process induced by Trypanosoma cruzi, which leads to myocarditis with reactive and reparative fibrosis. CCC progresses with myocardial perfusion abnormalities and histopathological events that affect cardiorespiratory fitness (CRF).

**Objectives:**

We evaluated the effects of aerobic physical training (APT) on myocardial perfusion and on morphological and functional impairments related with inflammation and fibrosis in Syrian hamsters with CCC. As a secondary objective, we analyzed the cross-sectional areas of the skeletal muscle.

**Methods:**

Hamsters with CCC and their respective controls were divided into four groups: CCC sedentary, CCC-APT, sedentary control and APT control. Seven months after infection, the animals underwent echocardiography, myocardial perfusion scintigraphy and cardiopulmonary exercise testing. Moderate-intensity APT was performed for fifty minutes, five times a week, for eight weeks. Subsequently, the animals were reassessed. Histopathological analysis was conducted after the above-mentioned procedures. The level of significance was set at 5% in all analyses (p<0.05).

**Results:**

CCC sedentary animals presented worse myocardial perfusion defects (MPD) over time, reduced left ventricle ejection fraction (LVEF) and showed more inflammation and fibrosis when compared to other groups (mixed ANOVA analysis). Conversely, APT was able to mitigate the progression of MPD, ameliorate inflammation and fibrosis and improve CRF efficiency in CCC-APT animals.

**Conclusions:**

Our study demonstrated that APT ameliorated cardiac dysfunction, MPD, and reduced inflammation and fibrosis in CCC hamster models. Additionally, CCC-SED animals presented skeletal muscle atrophy while CCC-APT animals showed preserved skeletal muscle CSA. Understanding APT's effects on CCC's pathophysiological dimensions is crucial for future research and therapeutic interventions.

## Introduction

Chagas disease is a neglected tropical disease that generates an annual global burden of US$ 627.46 million in healthcare costs^1^ and affects six to eight million people worldwide.^2^ The disease is caused by the protozoan *Trypanosoma cruzi* (*T. cruzi*) and most of the infected subjects remain asymptomatic throughout their lives (indeterminate form). However, about 40% progress to clinical forms (cardiac or digestive) 10 to 30 years after the initial acute infection.^3^ Chronic Chagas cardiomyopathy (CCC) is the most severe and progressive nonischemic cardiomyopathy in Latin America.^4^ CCC manifests with electrocardiography disturbances, wall motion and perfusion abnormalities, thromboembolic events, and heart failure, which may lead to sudden death.^5^

The main pathogenetic mechanisms of CCC are related to parasite persistence and immune-mediated myocardial injury, microvascular disorders and cardiac dysautonomia.^6^ In the chronic phase, the distinctive high load parasitemia observed in the acute phase is lessened by the immune response into a low-grade but persistent infection.^7^ This low-intensity inflammatory process, thus, leads to multifocal myocarditis with mononuclear infiltrates, myocytolytic lesions, necrosis, microvascular derangement, mitochondrial dysfunction, cardiomyocyte hypertrophy, increased areas of interstitial and perivascular fibrosis and cardiac remodeling.^1,8–10^

Myocarditis and reparative fibrosis are hallmarks of CCC.^6^ The myocardial inflammatory infiltrate is mainly composed by T cells and macrophages.^11^ Moreover, there is also an increased expression of inflammatory cytokines interferon-gamma (IFN-γ), tumor necrosis factor-alpha (TNF-α), interleukin-6 (IL-6)^12,13^ and chemokines.^11,14^ Additionally, persistence of *T. cruzi*, leading to continued production of INF-γ and TNF-α, stimulates oxidative and nitrosative stress that damage cardiomyocytes’ mitochondria, cause mitochondrial dysfunction and compromise energy production pathways.^15,16^ In addition to the above mentioned alterations that are associated with the poor prognosis of the disease, coronary microvascular dysfunction and the following myocardial perfusion disturbances may be an early mark of disease progression.^17,18^ These disturbances are also related to myocytolytic necrosis and development of cicatricial injuries linked with areas of regional transmural fibrosis.^19^

Beside the myocardial damage, chronic *T. cruzi* infection can also affect the skeletal muscle. The main alterations include skeletal myositis, with mononuclear exudate,^20^ necrosis of fiber cells,^21^ muscle fiber disorganization and atrophy,^20^ vasculitis and fibrosis,^22^ capillary damage with thickening and reduplication of the basement membrane, smaller skeletal muscle cross-sectional area (CSA),^23^ and muscle denervation.^24^ CCC subjects may also present occluded capillaries and more glycolytic and less oxidative activity in the skeletal muscle.^25^ Together, these alterations may affect oxygen extraction, reduce oxygen delivery and lead to functional impairment.^25^

One of the main factors associated with morbidity and mortality in CCC is the disease progression^26^ that affects cardiorespiratory fitness (CRF)^27^ and quality of life.^28^ Currently, the treatment for CCC is mainly based on controlling the symptoms of the disease.^1,5,29^ However, despite the scarcity of prospective randomized trials,^30^ some studies have already observed the benefits of exercise training in these subjects, with major improvements in CRF.^31–34^ The latest guideline regarding CCC^1^ has stated that physical activity improves many clinical parameters, but the benefits for this population have not yet been fully addressed. Hence, this approach has "conditional" grade recommendation and a "B" level of evidence.^1^

Improvements in peak VO_2_ have been observed after exercise training in subjects with CCC.^30,31^ Other benefits comprise improvements in left ventricular ejection fraction (LVEF), muscle respiratory strength,^33^ cutaneous microvascular function^35^ and in quality of life.^30^ In experimental studies, exercise training before or after acute experimental *T. cruzi* infection modulated inflammatory reaction and improved resistance against *T.cruzi*,^36^ induced reduction in serum activities of creatine kinase and creatine kinase-myocardial band (CK-MB),^37^ and reduced cardiac fibrosis.^38^ In CCC, only a study demonstrated that low-intensity aerobic exercise improves morphological and morphometric parameters of the right and left ventricles.^39^

In CCC experimental investigations, various animal models have been used.^8,9,40–43^ Among these, the Syrian hamster murine model is the one that develops CCC resembling the human infection with the typical natural history, and histological, structural and functional changes of the disease with a more adequate timeline for research studies.^10,43,44^ Despite this evidence, none of these investigations have focused on the effects of aerobic exercise on myocardial perfusion, histopathological alterations (inflammation and fibrosis) and skeletal muscle changes in Syrian hamsters with CCC. Considering the several cardiovascular adaptations,^45–47^ we believe that aerobic exercise may lead to improvements in the main pathogenetic mechanisms involved in the disease progression: myocardial perfusion defects (MPD) and inflammation. Therefore, this study aimed to evaluate the impact of aerobic physical training (APT) on myocardial morphological, functional and perfusion changes, correlating these variables with myocardial inflammation and fibrosis in Syrian hamsters with CCC by using high-resolution imaging *in vivo*. In addition, as a secondary objective, we aimed to analyze the cross-sectional areas of the skeletal muscle of this experimental model.

## Methods

### Study design

In this experimental study, female Syrian hamsters (*Mesocricetus auratus*), aged 12 weeks, obtained from Anilab (*Animais de Laboratório Criação e Comercio* Ltda, Paulínia/SP, Brazil) were kept in a temperature-controlled room with free access to water and standard food and were submitted to a 12-hour light-dark cycle. The animals were kept in a cage following Brazilian National Council for the Control of Animal Experimentation (CONCEA) recommendations, with an area/animal > 122.5 cm² and environmental enrichment appropriate for the species. All the procedures were conducted during the light phase of the light-dark cycle.

The animals were randomly allocated using a randomization generator (www.random.org Random.org, Dublin, Ireland). Initially, they (n=60) were allocated into infected and non-infected groups. After the acute phase of infection, the surviving animals [infected (n=37), non-infected (n=16)] were allotted into four experimental groups – APT and sedentary: CCC-APT (n=22), CCC-SED (n=22); and two control groups also divided into APT and sedentary: CT-APT (n=8) and CT-SED (n=8). The procedures were conducted by a researcher, blind to the groups and unaware of the treatment.

The animals were infected intraperitoneally with 3.5x10^4^ trypomastigote forms of the Y strain of *T. cruzi,* while the control group was inoculated with saline solution (0.4 ml). Seven months after infection (Figure 1), the animals were submitted to two-dimensional echocardiography (2-D ECHO), single photon emission tomography (SPECT) with ^99m^Tc-Sestamibi (RPHKARDIA, Porto Alegre, Brazil) and cardiopulmonary exercise testing (CPET). The image assessments lasted one week with two to three days between each test. The CPET lasted another five days for climatization and performance of the tests. The exercise training started two days after CPET. Eight weeks after the intervention period (APT), the animals underwent the same assessments (CPET, ECHO-2D and SPECT), followed by euthanasia [ketamine (Vetbrands, Jacareí, São Paulo, Brazil) and xylazine (Bayer, São Paulo, Brazil), 160 mg/Kg and 10 mg/Kg, respectively] and collection blood samples collection to confirm chronic Chagas disease.^48^ The confirmation of chronic *T. cruzi* infection was carried out with a Western blot assay to detect anti-*T. cruzi* antibodies in the sera of the infected animals as previously described.^48,49^ Cardiac tissue and skeletal muscle samples were collected for histopathology.

**Figure 1:** Study timeline. After the experimental infection with the Y-strain of *T. cruzi* and the follow-up period of seven months for the development of the disease, the surviving animals and the control group underwent two-dimensional echocardiogram (ECHO-2D), SPECT with ^99m^Tc-Sestamibi and cardiopulmonary exercise testing (CPET). Afterwards, they were allocated in four groups [CT-SED (n=6), CCC-SED (n=16), CT-APT (n=8), CCC-APT (n=12)]. Subsequently, the APT groups were submitted to eight weeks of aerobic physical training (APT) and reassessed. Lastly, they were euthanized, and the heart and skeletal muscle were collected for the histopathological analysis. Illustration created with BioRender.com

The study was conducted in compliance with the recommendations of CONCEA after due approval from the Ethics Committee on Animal Use – CEUA (N°229/2019) of our institution.

### Two-dimensional echocardiography

Doppler echocardiography was performed using a dedicated high-resolution two-dimensional echocardiography (ECHO 2-D) system for small animals Vevo® 2100 (*Visual Sonics Inc*., Toronto, Canada) with a 30 MHz frequency linear transducer. After sedation with ketamine and xylazine (80 mg/Kg and 5 mg/Kg, respectively), the animals were shaved and placed in left lateral decubitus. Images from the parasternal long and short axis of the left ventricle were obtained. Two-dimensional images were used to assess LVEF, left ventricular (LV) end-diastolic and LV end-systolic volumes in the parasternal long-axis view as previously described.^50^

### Myocardial perfusion imaging

To assess myocardial perfusion at rest, high-resolution SPECT images were acquired with ^99m^Tc-Sestamibi, using a gamma camera (BrightView XCT; Philips Medical Systems Inc., Cleveland, OH) adapted with an image acquisition system with a "pinhole" collimator of 1.5 mm opening positioned parallel to a rotational support for the animal, as previously described elsewhere.^48,51^

Briefly, under isoflurane anesthesia^52^ (Isoforine, São Paulo, Brazil), the animals received 555 MBq of MIBI through the sublingual vein and were allowed to awake. After 90 minutes, the hamsters were re-anesthetized with a combination of ketamine (80mg/kg) and xylazine (5mg/kg) and SPECT images were acquired. Images, collected in upright position, were reconstructed using a three-dimensional ordered-subset expectation maximization algorithm (3D-OSEM, four subsets and 10 interactions). Radiotracer accumulation in the myocardium was analyzed semi-quantitatively using polar maps generated by software MunichHeart® (MunichHeart software, Technical University Munich, Munich, Germany). The MPD were considered significant if they were higher than 5% of the left ventricle.^48^

### Cardiopulmonary exercise testing

Previously to CPET, the animals were familiarized with the treadmill for five consecutive days with incremental velocities and fixed inclination (5°).^53^ For the evaluation of the cardiorespiratory fitness of the animals, oxygen consumption (VO_2_) and carbon dioxide production (VCO_2_) were continuously measured by open-flow indirect calorimetry on a treadmill with speed and incremental inclination (Panlab, Harvard Apparatus, Spain). VO_2_ was expressed in units adjusted to the animal's size (mL.kg^-0.75^.min^-1^) and peak VO_2_ was defined as the highest VO_2_ value measured during the test before exhaustion. The VO_2_ at anaerobic threshold (VO_2AT_) was determined as the oxygen consumption at which a linear relationship between VCO_2_ and VO_2_ was lost during progressive treadmill exercise associated with an abrupt increase in respiratory exchange ratio.

The stress protocol has been described before.^54^ Concisely, the protocol consists in increasing speed and inclination of the treadmill at each stage. The first three stages lasted two minutes with a five-degree increment in each stage. From stage four onwards, the duration was of one minute, and inclination was maintained at 15 degrees. The speed started at 15 cm/s and increased 5 cm/s each stage until stage six. From that, the speed increased 1.67 cm/s each stage until the animal's exhaustion. The criteria for interrupting the test included the animals staying for five seconds or longer on the electrical stimulation grid or staying for 10 seconds on the distal end of the treadmill.^53,54^

### Aerobic physical training

After basal evaluation (ECHO 2-D, SPECT and CPET), the animals underwent APT on a treadmill (Gaustec Magnetismo, Nova Lima, Minas Gerais, Brazil), following an 8-week protocol adapted from previous work.^53^ APT was performed 5 times a week for 50 minutes at moderate intensity (50% of the peak velocity defined by the CPET and 5% treadmill grade) in the same period of the day. The time, speed and grade were progressively increased in the first two weeks until the prescribed intensity was reached. To ensure similar handling and exposure to APT animals, the sedentary animals were submitted to two minutes of treadmill running five days per week, with the same running speed as APT animals. After the training period, the evaluation methods were repeated and the animals euthanized for tissue collection for histopathological study.

### Histopathological analysis

For histopathological analysis, transverse tissue slices (5 μm thick) were obtained from three sections of the heart (basal, mid-ventricular and apical), maintaining the orientation for the topographic correlation with *in vivo* images as previously described.^10^ After progressive dehydration, the tissue was fixed in paraffin and samples from each ventricular section were stained with hematoxylin-eosin and picrosirius-red to quantify inflammation and fibrosis, respectively. After that, digital microscopy images (40× objective lens) of endocardium, myocardium and epicardium from each LV segment were taken using BX51 Olympus microscope (Olympus; Tokyo, Japan) equipped with a Q-color 5 camera (Olympus America, Center Valley, Inc., USA). For picrosirius-red slices, polarized light microscopy photographs were also taken to quantify type I and type III collagen, by identifying bright red-yellow and green fibers, respectively. Subsequently, they were analyzed using the Aperio ImageScope (version 12.4.6, Leica Biosystems Imaging, Inc., USA) and Image Pro Plus 32 (version 4.5.0.29; Media Cybernetics, Inc., Maryland, USA) software.

Inflammation was quantified by counting the number of mononucleated cells per field (cells/mm^2^). Picrosirius red staining defined the interstitial fibrosis as the percent (%) of total area with caution to exclude perivascular fibrosis. To analyze the histopathological alterations, the left ventricle was divided into 16 segments: basal (anterior, anteroseptal, inferoseptal, inferior, inferolateral, anterolateral), mid-ventricular (anterior, anteroseptal, inferoseptal, inferior, inferolateral, anterolateral) and apical (anterior, septal, inferior, lateral).

Transverse skeletal muscle sections (5 μm thick) from medial portion were fixed in paraffin and, later, were stained with hematoxylin-eosin to quantify the fiber CSA. Histological images of the muscle middle portion (bar=100 μm, 20x) were analyzed using the softwares Aperio ImageScope (version 12.4.6, Leica Biosystems Imaging, Inc., USA) and ImageJ Fiji software (version JAVA 1.8.0_322., National Institutes of Health, Bethesda, Maryland, USA). For the CSA analysis, about 200 muscle fibers were measured per sample. Additionally, we only used a representative number of animals [CT-SED (n=4), (C) CCC-SED (n=6), (D) CT-APT (n=4) and (E) CCC-APT (n=5)] from each experimental group.

### Sample size calculation

The sample size was calculated using OpenEpi online software (Open Source Epidemiologic Statistics for Public Health, version 3.1, Atlanta, GA, USA), and the criteria used to define the sample size were based on previous studies.^10,55^ A 10% reduction in the perfusion defect between infected groups at the end of the treatment was assumed, with two-tailed alpha of 0.05, 1-β= 0.8. The estimated sample size for this study was 13 animals in each infected group and eight animals in the control groups. As the study includes four groups (two controls and two infected groups), the total number was 42 animals. However, a loss of 40% was considered due to the aggressiveness of the parasitemia in the groups of infected animals. We also considered that only 30 to 50% of chronically infected animals develop Chagas cardiomyopathy.^12^ Therefore, the total number of animals used in this study was 16 control animals (CT-SED = 8 animals; CT-APT = 8 animals) plus 44 infected animals (CCC sedentary = 22 animals; CCC APT = 22 animals), totaling 60 animals.

### Statistical analysis

Continuous variables are reported as mean ± standard deviation and categorical variables are expressed as absolute (n) and relative (%) frequency. The Kolmogorov–Smirnov Test was used to verify Gaussian distribution of the variables. One-way ANOVA analysis of variance was used for simultaneous comparison of the four experimental groups at baseline and for histopathological analysis of inflammation and fibrosis. The mixed ANOVA (mixed ANOVA or split-plot factorial ANOVA) for repeated measures was used to verify the interaction (main effect) between the experimental groups (between-subject effect) and the time (within-subject effect) for the effect of APT on ECO-2D, SPECT and CPET variables. In case of statistically significant interactions, the Bonferroni post hoc multiple comparisons were conducted.

The statistical analysis and the graphs were made using GraphPad Prism software (version 9.0.0; GraphPad Software, San Diego, California, USA). The level of significance was set at 5% in all analyzes (p<0.05).

## Results

In the acute phase of infection (up to 35 days after infection), a mortality rate of 16% (n=7) was observed. Thirty-seven infected animals and 16 controls underwent baseline evaluations (7 months after infection). During baseline evaluations, three infected animals died due to anesthesia. Another six infected animals (CCC-SED, n= 4 and CCC-APT, n= 2) and 2 controls died during the intervention period. No animal died while performing APT.

### APT ameliorates LV remodeling and LV systolic and perfusion dysfunction

The results of Echo-2D and myocardial perfusion SPECT at baseline and after reassessments post-APT are presented in Table 1. Regarding LVEF, a significant interaction between the time and experimental groups was observed. LVEF had an important reduction only in CCC-SED group. The CCC-APT was the only group that presented LV diastolic dilation over time. However, this group did not present increase in LV mass or LV systolic diameter. Regarding the MPD, the CCC-SED group presented worsening of perfusion defects over time.

**Table 1:** Echocardiography and myocardial perfusion SPECT data from the experimental groups before and after the aerobic physical training

Variables	CT-SED (n=6)	CCC-SED (n=16)	CT-APT (n=8)	CCC-APT (n=12)	p Values
**LVEF (%)**					
Pre	52.93 ± 1.49	45.49 ± 8.84	49.86 ± 2.93	43.74 ± 9.92	
Post	52.47 ± 3.59†	39.69 ± 7.80*	47.28 ± 5.67†	42.46 ± 4.47	0.007
**LVDD (mm)**					
Pre	7.71 ± 0.51	7.57 ± 0.70	7.73 ± 0.34	7.71 ± 0.43	
Post	7.96 ± 0.56	7.99 ± 1.00	8.22 ± 0.50	8.34 ± 0.60*	0.030
**LVSD (mm)**					
Pre	5.68 ± 0.43	5.68 ± 0.94	5.99 ± 0.40	6.13 ± 0.54	
Post	6.08 ± 0.53	6.06 ± 1.01	6.47 ± 0.81	6.80 ± 0.74	0.050
**LV Mass (mg)**					
Pre	519.19 ± 89.55	549.85 ± 96.73	532.02 ± 98.15	541.89 ± 84.71	
Post	667.78 ± 125.04*	686.99 ± 152.06*	735.47 ± 170.16*	622.15 ± 129.83	0.014
**MPD (%)**					
Pre	2.45 ± 1.69	4.64 ± 3.45	3.08 ± 2.56	4.81 ± 3.52	
Post	3.31 ± 2.44†	8.29 ± 7.06*	2.56 ± 3.43	6.30 ± 3.38	0.010

Data are reported as mean ± standard deviation. APT: aerobic physical training, CCC: chronic Chagas cardiomyopathy, CT: control, SED: sedentary, LVEF: left ventricle ejection fraction, LVDD: left ventricle diastolic diameter, LVSD: left ventricle systolic diameter, LV mass: left ventricle mass, MPD: myocardial perfusion defect, SPECT: single-photon emission computerized tomography; mixed ANOVA for repeated measures;

* p<0.05 vs. baseline of the same experimental group,

† p≤0.05 vs. post-treatment CCC-SED, Bonferroni test for multiple comparisons.

Animals presenting significant MPD (> 5% of LV) were detected in both infected groups at baseline, with five animals (36%) in the sedentary and three (25%) in the exercise group. After follow-up, we observed an increase in the size (p< 0.05) and number of animals (n=10, 67%) with MPD in the CCC-SED group while in the APT group we noticed a smaller increase in the size (p>0.05) and number of animals with perfusion abnormalities (n= 5, 42%).

Figure 2 shows an example of an animal from the CCC-SED group that presented LV systolic diameter (LVSD) dilation associated to MPD increase over time (Figures 2 A and B) in comparison with an animal from the CCC-APT with preserved LV function and morphology and no increase in MPD (Figures 2 C and D).

**Figure 2:** Effects of APT in cardiac function and perfusion. (A) and (B) show representative images of ECHO-2D and SPECT with MPD of an animal from CCC-SED (n=16), and (C) and (D) of an animal from CCC-APT (n=12); APT: aerobic physical training, CCC: chronic Chagas cardiomyopathy, SED: sedentary, MPD: myocardial perfusion defect.

### APT increases the efficiency of cardiorespiratory fitness in CCC

CPET variables are shown in Table 2. Besides the animals did not present significant improvements in the oxygen consumption at the peak of exercise or at the anaerobic threshold, CT-SED was the only group that did not present increase in the time until the exhaustion after the follow-up period. However, greater improvements were observed in groups that underwent exercise training. Although CCC-APT presented an increase in VO_2AT_, and a decrease was observed in CCC-SED, this difference did not reach statistical significance.

**Table 2:** Cardiopulmonary exercise testing data from the experimental groups before and after the aerobic physical training

Variables	CT-SED (n=6)	CCC-SED (n=16)	CT-APT (n=8)	CCC-APT (n=12)	p Values
**Time until exaustion (s)**					
Pre	464.67 ± 32.47	445.21 ± 72.19	494.00 ± 71.25	481.20 ± 76.57	
Post	557.50 ± 91.24	539.64 ± 94.04*	686.78 ± 109.59*†	687.20 ± 141.67*†	0.043
**VO_2peak_ (mL.Kg.min)**					
Pre	38.50 ± 3.46	42.70 ± 5.45	42.11 ± 4.40	40.32 ± 5.08	
Post	43.04 ± 4.90	43.75 ± 4.42	43.33 ± 3.43	45.60 ± 5.65*	0.254
**VO**_2@AT_ **(mL.Kg.min)**					
Pre	31.22 ± 6.79	33.06 ± 5.48	30.12 ± 4.54	30.56 ± 3.25	
Post	30.90 ± 6.25	28.95 ± 5.86	33.44 ± 3.96	34.39 ± 4.06†	0.759

Data are reported as mean ± standard deviation; APT: aerobic physical training, CCC: chronic Chagas cardiomyopathy, CT: control, SED: sedentary, VO2: oxygen consumption; VO2AT: oxygen consumption at anaerobic threshold. Mixed ANOVA for repeated measures;

* p<0.05 vs. baseline of the same experimental group,

† p≤0.05 vs. post-treatment CCC-SED, Bonferroni test for multiple comparisons.

### APT ameliorates myocardial inflammation and fibrosis and preserves skeletal muscle CSA

Myocardial inflammation was higher in animals from CCC-SED when compared to CT-SED, CT-APT and CCC-APT (1.61±0.63 vs 0.37±0.12 vs 0.7±0.2 vs 0.93±0.2 cells/mm^2^, respectively, p< 0.001). No significant difference in total fibrosis (p> 0.05) was observed. However, the CCC-SED group, but not the CCC-APT group, presented higher type I collagen expression when compared to control groups (p<0.05) (Figure 3).

**Figure 3:** Bar graphs showing the results of (A) myocardial inflammation, (B) total fibrosis, (C) type I collagen and (D) type III collagen. Representative histopathological samples of (E) CT-SED (n=6), (F) CCC-SED (n=16), (G) CT-APT (n=8) and (H) CCC-APT (n=12) tissues stained with hematoxylin & eosin and picrosirius red. APT: aerobic physical training, CCC: chronic Chagas cardiomyopathy, CT: control, SED: sedentary. Bar= 50 μm, 40x magnification.

Furthermore, skeletal muscle atrophy was confirmed by the reduction in CSA. CCC-SED presented skeletal muscle atrophy that was normalized by exercise training in CCC-APT group, as observed in the Figure 4. The CCC-SED animals frequently presented skeletal muscle inflammation that was less observed and less intense in the CCC-APT animals.

**Figure 4:** (A) Bar graphs showing the quantitative analysis of the gastrocnemius muscle cross-sectional area (CSA) of animals from the infected and control groups. Representative gastrocnemius muscle histopathological images taken from (B) CT-SED (n=4), (C) CCC-SED (n=6), (D) CT-APT (n=4) and (E) CCC-APT (n=5) groups. Diffuse mononuclear inflammatory infiltrate can be seen in CCC-SED (C). APT: aerobic physical training, CSA: cross-sectional area, CCC: chronic Chagas cardiomyopathy, CT: control, SED: sedentary. Bar=100 μm, 20x magnification.

## Discussion

The present study investigated the effects of APT in an experimental model of CCC in Syrian hamsters by using *in vivo* high-resolution imaging techniques and CPET. The main findings of this study were that APT mitigated the progression of MPD, LV remodeling and LV systolic deterioration, in addition to improving the efficiency of CRF. Furthermore, APT ameliorated myocardial inflammation and fibrosis and improved the CSA of skeletal muscle.

In our study, the CCC-SED group presented worsening of perfusion defects over time. Progressive MPD as a pathophysiological mechanism of the disease has been observed previously both in experimental^48,55^ and clinical^56,57^ scenarios of CCC. Moreover, some works have hypothesized that myocardial perfusion abnormalities may precede LV systolic deterioration.^19,58^ Therefore, therapy aiming at improving myocardial perfusion may prevent the progression in cardiac damage. In our study, we did not observe improvements in MPD after exercise intervention; however, the perfusion defects did not increase significantly after the exercise in contrast to the infected sedentary group.

To the best of our knowledge, this is the first work using this strategy to treat MPD in CCC. A recent pilot study investigated the effects of aerobic exercise over MPD in patients with primary microvascular angina, a disease with a similar mechanism to myocardial perfusion disturbances.^59^ The authors observed a significant reduction in myocardial perfusion disturbances associated with improvements in VO_2peak_ and quality of life. The exercise also promoted benefits in myocardial perfusion in coronary artery disease^60,61^ and heart failure^62,63^ patients. The mechanisms by which exercise training promotes myocardial perfusion are probably multifactorial and several have been described such as improved endothelial function,^64–66^ coronary vascular adaptations^67^ and enhanced collateralization.^68^ Moreover, reduction in inflammation and improvements in autonomic and neurohormonal balance may contribute to it.

Regarding other treatments for myocardial perfusion in CCC, Tanaka et al.^55^ have used dipyridamole, a coronary vasodilator agent, to improve MPD in female Syrian hamsters. The authors observed a significant MPD improvement in the treated groups compared to the control groups. However, the treatment did not interrupt the progressive LV dysfunction. According to the authors, despite improvements in perfusion, the disease progressed because the treatment had no benefits on myocardial inflammation, which remained similar in the two infected groups. Recently, Tanaka et al.^17^ investigated the same animal model with CCC treated with pentoxifylline. The authors reported that this treatment reduced the inflammation and MPD but did not prevent the progression of LV systolic dysfunction. They hypothesized that maybe the period of intervention (six months after the infection) was not enough to improve LV systolic dysfunction, but did reduce inflammation and MPD as they occur in the early stages of the disease, and precede the LV systolic dysfunction. Another study, using verapamil (calcium channel blocker) plus aspirin (non-steroidal anti-inflammatory drug), has demonstrated significant improvements in MPD and quality of life in CCC patients.^69^ Unfortunately, the authors did not assess the systolic function or inflammation after the intervention. The beneficial effects of verapamil in CCC have also been demonstrated in *T. cruzi* infected mice.^9,70^

Chronic *T. cruzi* infection in humans generally leads to a more aggressive cardiovascular damage, with higher amount of fibrosis and more severe ventricular remodeling, in males than in females.^71^ In animals, peak parasitemia and disease progression seem more homogeneous in females.^55,72^ As several reports^10,17,21,43,48,55^ have successfully used female Syrian hamsters when investigating CCC, we chose this murine model to study the disease. Our results are in accordance with previous research^10,43^ in which Syrian hamsters developed the disease resembling human CCC. LV dysfunction, a marker of disease severity, was observed in the first assessment seven months after the parasitic infection, when the LVEF was already reduced. Similar findings were described by Ribeiro et al.,^73^ where LVEF and LV end-systolic diameter were deteriorated at six months of disease onset and progressed later in this same animal model. However, we observed that the exercise training interrupted LV dysfunction progression. Only few clinical trials have investigated the LV function and morphology after exercise.^31,74^ None of the above-mentioned studies documented improvements in heart function or morphology after the exercise. However, the studies were small, with different profiles of patients and, therefore, more studies with more patients and longer follow-up periods are necessary to investigate whether exercise training may improve or interrupt cardiac deterioration in CCC.

Furthermore, in the histopathological analysis we noticed that the infected sedentary animals showed a higher extent of inflammatory infiltrates with clusters of mononuclear inflammatory cells and areas with extensive fibrosis when compared to other animals. The role of inflammation and fibrosis triggering perfusion abnormalities was already reported by other researchers. Bilate et al.^43^ found a statistically significant correlation between myocarditis and interstitial fibrosis. The authors suggested that the cardiac inflammatory infiltrate was likely responsible for this progressive tissue injury, and consequent remodeling and extensive fibrosis. In addition, Oliveira et al.^48^ reported that these perfusion disturbances may be localized in regions with viable myocardium and reduced perfusion secondary to inflammation. Therefore, we demonstrated that APT performed at moderate intensity five times a week for eight weeks were able to ameliorate inflammation and fibrosis. Myocardial inflammation is the main histopathological characteristic of CCC, and therapies targeting it have demonstrated promising benefits on the cardiac function.^1,75^

Cardiac fibrosis is considered an independent predictor of adverse outcome in this cardiomyopathy.^76^ Fibrosis also plays an important role in the impairment of cardiac performance and heart dilation.^77^ Ramírez et al.^21^ reported heart dilation (mainly in the apex) and mural thrombosis in Syrian hamsters with CCC. In our study, we observed diastolic dilation in the CCC-APT group over time. However, we hypothesized that this increase was a physiological adaptation to exercise, since no difference was observed in LVEF or fibrosis between this group and uninfected animals. The amount of type I collagen was higher in the CCC-SED group, but we did not notice statistically significant difference between the groups with Chagas disease that underwent exercise.

CCC patients may also present skeletal muscle abnormalities.^20^ In our study we analyzed samples from gastrocnemius muscle; the CCC-SED group presented atrophy and in the CCC-APT group, muscle atrophy was normalized after exercise training. Although some previous researches^20–25,78–81^ have investigated skeletal muscle abnormalities after *T. cruzi* infection, just a small number have analyzed the chronic phase and none have evaluated the muscle CSA in the chronic phase after APT. Concerning experimental studies, Silva et al.^80^ observed myositis with mononuclear exudate and fibrosis in the diaphragm, intercostal and psoas muscles of rabbits after six months of infection.^80^ Weaver et al.^22^ evaluated the quadriceps muscle of mice in the early (2 – 4 months) and late (9 – 10 months) chronic phase. The authors reported few *T*. cruzi parasites in the muscle, inflammation, necrotizing vasculitis, vascular fibrosis, endomysium fibrosis and gait abnormalities (including avoidance of weight-bearing on any limb, foot dragging, and even paresis).^22^ Ramírez et al.^21^ evaluated the skeletal muscle (muscle not specified) of Syrian hamsters and reported focal myositis and necrosis. Souza et al.^78^ noticed an upregulated expression of chemokines and that the number of inflammatory cells remained elevated in skeletal muscle (muscle not specified) of mice at all time points evaluated from acute to chronic phases. The reports in human biopsy studies of CCC showed capillary damage and smaller skeletal muscle CSA (vastus lateralis muscle);^23^ inflammation, muscle fiber disorganization and atrophy (biceps muscle);^20^ denervated atrophic muscle fibers (gastrocnemius muscle);^24^ capillary occlusion, higher percentage of muscle fibers with lower oxidative capacity and enhanced percentage of fibers with more glycolytic capacity (vastus lateralis muscle).^25^

Regardless of the well-established benefits of aerobic exercise in cardiac patients,^45,82^ only a few clinical studies have investigated APT in the treatment of Chagas cardiomyopathy.^30^ Lima et al.^32^ observed improvements in cardiorespiratory fitness (increases in VO_2peak_, exercise time and six-minute walk test distance) and no adverse effects of exercise in the treated patients. Articles from PEACH study^29,31,35,83^ addressed the effects of exercise training in patients with Chagas cardiomyopathy. The studies observed major improvements in CRF in patients with LV dysfunction and heart failure. Moreover, the continued beneficial effects of the exercise were still noticed at three^48^ and six^31^ months of follow-up. The exercise performed for six months also improved cutaneous vascular responsiveness to reactive hyperemia.^35^

Our study also detected positive effects of APT in CCC, with increase in exercise capacity in APT groups after eight weeks of training (moderate intensity, five days/week, 50 minutes). Regarding previous experimental studies, only six^36–38,84–86^ have investigated the effects of APT in other phases of Chagas disease and only one^39^ has addressed the role of aerobic exercise on established CCC. Schebeleski-Soares et al.^86^ conducted an 8-week treadmill exercise (moderate intensity, five days/week, progressive speed and duration) before *T. cruzi* infection in mice. Soares et al.^37^ performed an 8-week treadmill training (moderate intensity, five days/week, incremental speed and duration) before *T. cruzi* infection in mice. Novaes et al.^85^ administered a 9-week treadmill protocol (moderate intensity, five days/week, progressive inclination, speed and duration) to Wistar rats before *T. cruzi* infection. Novaes et al.^84^ also used the same protocol in a further investigation with Wistar rats (9-week treadmill exercise, moderate intensity, five days/week, progressive inclination, speed and duration) before *T. cruzi* infection. Lucchetti et al.^36^ performed a 9-week treadmill protocol (workload intensity defined by the maximal lactate steady state, five days/week, 60 minutes)^87^ before *T. cruzi* infection in mice. Pedra-Rezende et al.^38^ studied the chronic indeterminate form of Chagas disease and used a 4-week treadmill protocol 140 days postinfection (moderate intensity, five days/week, progressive increase in speed, 60 min). Finally, Preto et al.^39^ used an 8-week swimming protocol (low-intensity aerobic exercise, five days/week, 30 minutes/day) to treat mice with CCC.

Although all these studies have used aerobic exercise as a non-pharmacological option to treat the animals, currently, just Preto et al.^39^ observed the effects of APT on CCC and showed that exercise improved morphological and morphometric parameters of the right and left ventricle. In the untrained animals, the authors observed impaired cardiomyocyte contractile function, more inflammation and higher amount of collagen, LV hypertrophy, decrease in the CSA of cardiomyocytes, microvascular changes and worsening of exercise tolerance.^39^ Considering this scenario, our results add knowledge about the effects of aerobic exercise on myocardial perfusion and histopathological alterations on the heart, and show that APT was able to preserve the skeletal muscle CSA. We highlight that this was the first experimental study in Syrian hamsters that used aerobic exercise in the treatment of CCC aiming to mitigate MPD, inflammation and fibrosis in the cardiac tissue. This was also the first study to assess the integrity of skeletal muscle CSA of CCC animals after eight weeks of aerobic exercise.

As a limitation, we studied only resting perfusion defects and it may be expected that ischemic perfusion defects would add value in the interpretation. However, it would be necessary to use inotropic positive drugs in anesthetized animals, which may interfere with the results. Moreover, our group has previously shown the correlation between MPD and inflammation in viable myocardium.^48,55^ Despite the positive results regarding the effects of APT, maybe a longer period of follow-up and exercise training would show more significant results regarding MDP. Additionally, we did not include in this paper the quantitative data regarding skeletal muscle inflammation. Future studies using exercise training in the management of CCC are necessary to strengthen the knowledge available.

Finally, this study supports the hypothesis that both MPD and inflammation contribute to systolic function deterioration in CCC, and exercise is an important strategy to minimize it. Exercise training has become a robust recommendation for most of the cardiac diseases. However, current guidelines do not explicitly recommend cardiac rehabilitation for CCC patients.^1,88^ Our results provide the foundation for further studies aimed at investigating the benefits of exercise intervention in CCC.

## Conclusion

Our study provides evidence that APT ameliorates cardiac dysfunction and MPD in a Syrian hamster model of CCC. Moreover, besides improving running performance, APT reduced inflammatory cell infiltration and fibrosis in the myocardium, indicating its potential as a therapeutic strategy for CCC. These findings highlight the importance of exercise training in mitigating the pathological progression of CCC and improving cardiac function. In addition to the significant findings related to cardiac alterations, it is noteworthy that we also observed skeletal muscle atrophy in hamsters with CCC and improvements in the muscle CSA after exercise training. Although these changes were not the focus of our investigation, they provided additional insights into the systemic complications associated with the disease. Therefore, a comprehensive understanding of the effects of APT on various pathophysiological dimensions of CCC remains an important aspect for future investigations.
